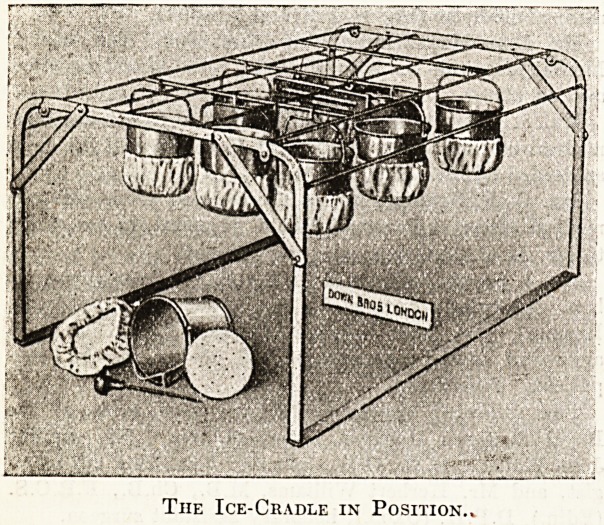# Institutional Needs

**Published:** 1913-02-08

**Authors:** 


### Institutional Needs.

### AN IMPROVED ICE-CRADLE.

The appliance, of which an illustration is appended, deserves immediate attention from the fact that it is the suggestion apparently of an institutional worker, Miss. K. C. Braidwood, matron of the Infectious Hospital, Mylands, Colchester, who placed her ideas in the well-known hands of Messrs. Down Bros., Ltd., 21-23 St. Thomas' Street, London, S.E., who now, as the makers, have placed it on the market. Designed, of course, to reduce the body temperature in cases of fever, its advantages to nurse and patient are summed up as follows: (1) The patient need not be moved; (2) economy of time and labour, as compared with such methods as baths, packs, etc.; (3) it can be easily managed by one nurse, and carried or stored, for the cradle folds flat when not in use. The apparatus consists of a light folding body cradle, to the top frame of

The Ice-Cradle in Position.

which are attached a thermometer and set of eight ice-pails. The bases of the latter are enveloped in flannel caps, which by absorbing the moisture due to condensation effectually prevent dripping. The pails can be detached singly, and the number in use at any time can thus be regulated. The method of use is as follows: The patient wears a long flannel gown and bed-socks, or is covered with a thin blanket, and has a water-bottle at the feet; the cradle is placed well up over the trunk of the patient, and the pails and thermometer put in position. Two blankets are arranged over the cradle to open in the middle, so that no inrush of air need accompany inspection of the thermometer. As the patient's temperature falls, one or more pails are taken out. The services required of the nurse in charge of the apparatus are: To note the cradle temperature every time the patient's temperature is taken and adjust the number of pails accordingly; to keep the pails replenished, which is only the work of a moment if a bowl of chipped ice and an empty bowl into which to drain the pails are brought to the bedside; and finally, of course, to see that the patient does not take ice from the pails to suck. Too many experiences of such appliances cannot be recorded, and readers are invited to record their opinions in this case.

## Figures and Tables

**Figure f1:**